# Is optometry ready for myopia control? Education and other barriers to the treatment of myopia

**DOI:** 10.12688/hrbopenres.12954.2

**Published:** 2020-04-23

**Authors:** Saoirse McCrann, Ian Flitcroft, James Loughman

**Affiliations:** 1 Centre for Eye Research Ireland, School of Physics and Clinical and Optometric Sciences, Technological University Dublin, Dublin, Ireland; 2 Children’s University Hospital, Dublin, Ireland; 3 African Vision Research Institute, University of KwaZulu Natal, Durban, South Africa

**Keywords:** myopia, myopia management, myopia control, attitudes, barriers, optometry

## Abstract

**Background: **With the increasing prevalence of myopia there is growing interest in active myopia control. However, the majority of progressive myopes are still prescribed single vision spectacles. This prospective study aims to elucidate the knowledge and attitudes of optometrists toward myopia control, and thereby identify perceived barriers to the implementation of a risk focussed model of myopia management.

**Methods: **A series of four focus group discussions were conducted involving optometrists in different settings and career stages.

**Results: **The key finding to emerge is a disconnect in myopia control knowledge and practices between academic optometrists, final year optometry students and clinicians in practice. Academic faculty believe the optometry curriculum should provide undergraduates with the clinical skills to practise myopia control, however, although students were knowledgeable in relation to myopia associated risk factors, some students had not yet undertaken any practical form of myopia control in their undergraduate degree. Furthermore, students may not receive hands-on myopia control experience during their supervised practice placement, as the majority of clinicians do not offer myopia control treatments, other than to communicate lifestyle advice to modify risk of myopia progression. Clinicians alluded to a lack of availability of myopia control interventions and identified a range of barriers relating to their training, clinical practice and public health challenges, financial, technological and other constraints that affect the implementation of such interventions.

**Conclusion: **﻿It appears optometrists have to yet embrace myopia control as a core element of the clinical eye care service they provide. Education, training, finance, and time restrictions, as well as limited availability of myopia control therapies were among the main perceived barriers to myopia control. This study revealed a distinct need for alignment between optometric training and the public health need for effective myopia control.

## Introduction

Myopia is the most common visual disorder in many parts of the world ^1, 2^, and is predicted to affect almost 5 billion people worldwide by 2050 ^1^. Children are becoming myopic at a younger age ^3^, with the average degree of myopia also continuing to increase in magnitude over time ^4, 5^. As high myopia is a leading cause of irreversible vision impairment and blindness ^6^, the increasing levels of myopia arguably represents one of the most important ophthalmic public health threats of our time, and has been recognised as one of the conditions requiring immediate priority by the World Health Organization’s Global Initiative for the Elimination of Avoidable Blindness ^7^.

While the causes of myopia are both genetic and environmental, the recent global increases in myopia prevalence are thought to primarily reflect changing environmental influences ^8^. There is evidence to suggest that children who spend more time outdoors are less likely to be or become myopic ^9^, with increased time outdoors demonstrated to reduce myopia onset by 11–34%, but with no consistent effect in slowing progression in eyes that are already myopic ^9^. Other factors, such as more time spent in education ^10^, and prolonged or continuous near work ^11^, have been associated with an increased risk of myopia development and progression.

There is also a growing body of evidence to support the idea that myopia risk can be managed and myopia progression controlled ^12^. A range of optical and pharmacological interventions, such as atropine eye drops (at varying concentrations), multifocal contact lenses, orthokeratology and defocus modifying spectacle lenses (DIMS) have been demonstrated to reduce myopia progression when compared with single vision spectacle lenses or placebo ^12– 14^. Studies have also indicated bifocal and progressive addition spectacle lenses can also slow the progression of myopia in children with near-point esophoria and larger accommodative lags ^15– 17^. Low dose atropine is not readily available as a licensed product in Europe but is accessible through clinical trials, with the recruitment of trial participants dependent on Optometrist referrals ^18– 20^. A number of contact lens options for managing myopia are also commercially available in Europe and are reported to slow myopia progression by approximately 30% to 60% ^13^. These include multifocal contact lenses, frequently fitted ‘off- label’ ^21^, novel myopia control lenses (becoming more readily available globally) ^22^, and orthokeratology contact lenses.

Despite these advances, the majority of progressive myopes are still prescribed single vision spectacles, especially in countries outside of Asia ^23– 26^. There is a scarcity of published literature that examines the possible reasons for the very limited uptake of active myopia management. A search of various databases including MEDLINE, EMBASE, Google Scholar, Scopus, the World Health Organization International Clinical Trials Registry Platform, and ClinicalTrials.gov during the preparatory stages of this study revealed only three research papers and a round table discussion that explored the knowledge and attitudes of eye care professionals toward myopia and its control ^23– 25, 27^. Rather than a lack of evidence for efficacy, the barriers appear largely attitudinal; eye-care practitioners consider the information required to implement myopia control techniques to be lacking, as well as reporting concerns about the safety, cost and availability of such measures ^23^.

Due to their specialist clinical skillset and their community base, optometrists are in a prime position to take the clinical lead on myopia control and prevention. Therefore, identifying the barriers that prevent optometrists from recommending or offering myopia treatment is essential, in order to inform future education needs and develop public health strategies designed to tackle the rising prevalence of myopia and its associated eye health complications. This prospective study was designed to elucidate the current knowledge gaps and attitudes of optometrists in Ireland toward myopia control, and thereby identify perceived barriers that may limit the transition to a risk-focussed management model of myopia in primary care practice ^28^, wherein eye care professionals would implement clinical management strategies designed to limit the risk of myopia onset, progression and development of future myopia-related complications for individual patients.

## Methods

A series of focus group discussions involving optometrists in different settings and career stages were conducted between October 2018 and November 2018 in Dublin, Ireland. An ‘invitation to participate’ email request was sent to Optometric practices for administrators to disseminate amongst their clinic based optometrists. Final year optometry students and optometry faculty at Technological University Dublin (TU Dublin) were contacted directly through the study investigator via email and invited to participate in the study. One focus group involved academic optometry faculty (n=6) at TU Dublin, one involved final-year students (n=11) of the undergraduate optometry programme at TU Dublin and two separate focus group discussions involved optometrists (n=6 in each) working in optometry practices across Ireland. Final year optometry students would complete undergraduate lectures in December 2018 and embark on their supervised clinical practice the following January. TU Dublin was chosen because it is the only third level institution to offer an undergraduate optometry course in the Republic of Ireland. During recruitment, it was made clear that no particular previous myopia control experience was required. No new issues were emerging during the second focus group with optometrists in practice, indicating that saturation of ideas had been achieved ^29^.

Key topics and pre-specified questions explored in the focus group discussions were informed by a review of the literature ( *see Extended data*) ^30^. Topic areas included participants knowledge of and attitude toward myopia and myopia control, myopia control education and training, and perceived barriers to myopia control practice. At the beginning of each session, registered optometrist participants were asked the year they qualified as an optometrist, their current job title and whether they had completed any postgraduate education in myopia management. The researcher (SMC, a qualified optometrist) made efforts to ensure that all participants had equal opportunities to engage in each focus group discussion. Focus group discussions were audio recorded, transcribed and coded according to key topics in preparation for analysis. Following transcription, recorded information was deleted and the data set was read to provide the researcher with a general overview of the discussion group outcomes. Subsequent analysis was used to identify patterns in the data which were coded into categories and labelled in a manner to capture the general meaning of the patterns identified. The collated data was then analysed thematically ^31^. Participants were informed of the nature of the study prior to obtaining verbal informed consent using audio recording. Ethical approval was obtained from the Research Ethics Committee at TU Dublin (reference 16–45) and all information was managed solely by the researcher to ensure confidentiality of responses. The consolidated criteria for reporting qualitative studies (COREQ) checklist for this study is deposited in TU Dublin’s ARROW repository ^32^.

## Results

The clinical experience of the practice-based optometrists, nine of whom were trained in Ireland and three in the United Kingdom, ranged from 1 to 11 years (mean= 5 ± 3 years). Two of these optometrists worked in independent practice and the remaining 10 optometrists worked in multiples or franchises. Academic participants were all trained in Ireland and were more experienced overall, ranging from 10 to 33 years since graduation (mean= 21± 11 years). Final year students were at completion of their fourth and final year of undergraduate training at TU Dublin and were due to leave the university environment to enter optometric supervised practice placement in the month following participation in the focus group discussion. Only one of the 29 participants, an academic from the optometry faculty, had previously completed any postgraduate training specific to myopia control. The duration of each focus group was approximately one hour. Focus groups were conducted in quiet classrooms with only the participants and researcher present for the discussion.

### Knowledge and attitudes toward myopia and myopia control

Participants in all focus groups were aware of the increasing prevalence of myopia worldwide. Concern about the vision threatening and public health implications associated with myopia was noticeably higher among academic participants compared to clinic based and student participants. Academic faculty and optometry students were knowledgeable in relation to environmental risk factors for myopia, and considered increased time spent outdoors important in reducing the risk of onset of myopia. In contrast, the general consensus among clinic based optometrists was that there is insufficient evidence relating to the benefits of outdoor activity in delaying myopia onset, with increased screen time frequently mentioned as the biggest environmental risk factor for myopia.

The overwhelming attitude from optometrists based in clinical practice was that their knowledge is too limited to offer myopia control treatment. One recent optometry graduate working in a multiple practice stated *“I don’t know anything about myopia control or myopia control contact lenses. I was never taught how to fit them,”* with another optometrist contributing *“the control of myopia is beyond our scope of practice”.*

Contrarily, academics felt failure to discuss myopia control or refer a progressive myope for myopia control treatment was verging on negligent and should be discouraged, with agreement around the opinion of one academic who commented *“you can’t deny treatment on the basis of your own limitation[s]”,* and another adding *“if the optometrist does not offer referral for myopia control, that is negligence”.*

Academics highlighted that increasing awareness of the importance of myopia control among the profession is necessary in order to exercise a culture of best practice, and suggested this should be driven by postgraduate education, widespread community education and optometrists with a focus on patient-centred care mentoring in clinical practice settings. One academic optometrist commented on the importance of parental education to influence a change in clinical practice behaviour, *“If parents are putting pressure on optometrists about myopia control, then that would make it happen”*.

A recurrent theme throughout the discussions was an eagerness among participants to learn about how to incorporate myopia control therapies into clinical practice. Clinic based optometrists and students felt they would benefit from a set of recommended guidelines and workshops on myopia control, along with more information on currently available myopia control interventions.

### Myopia control in practice

A major theme to emerge from the focus group discussions was a clear disparity in the approach to myopia control between academic optometrists, final year optometry students and clinicians in practice. Academic faculty felt it was unacceptable to continue to treat progressive myopes with single vision spectacles, and considered themselves competent in managing progressive myopia; either by offering myopia control therapy such as multifocal contact lenses or orthokeratology, or by referral to a practice offering myopia control. Academics believed the optometry curriculum should provide undergraduates with the clinical skills and knowledge to practise myopia control, with consensus around the opinion voiced by one academic optometrist that *“In terms of educating current graduates, yes there is a lot done, the undergraduates should be experts on myopia control, but we don’t do a lot in terms of post graduate education”*.

The final-year undergraduate students, however expressed an almost universal lack of confidence in their current ability to practise myopia control, with only one student indicating they would initiate any form of myopia control therapy for a child exhibiting progressive myopia. Students acknowledged there was substantial emphasis on myopia control theory in the optometry curriculum, but felt the content was not structured or organised, as it was interspersed between various modules. The general consensus from undergraduates was that their exposure to implementing myopia control techniques in their primary care and contact lens training clinics was dependent on their supervisors interest in or ability to practise myopia control, with some students never having undertaken any practical form of myopia control, other than to communicate lifestyle advice to modify risk of myopia progression. One undergraduate student commented *“I know the theory but I have little practical experience. There is a lot of variation between supervisors too, therefore some students get to practice myopia control more than others”.*

Although clinical practice experience in paediatric optometry as well as fitting rigid gas-permeable (RGP) and multifocal soft contact lenses are core components of optometry training, the ability to successfully demonstrate a myopia control contact lens fit is not prioritised as a core competency in the undergraduate training programme at TU Dublin, even though fitting a soft contact lens for myopia control requires the exact same skill as routine single lens or multifocal lens prescribing ^33, 34^.

Clinic based optometrists engaged in very little discussion when asked their management strategy for the control of progressive myopia. Two participants indicated they would give *“The full minus correction as opposed to under minusing”* and *“recalls of shorter periods if you think they are progressing quite fast”* but did not offer any myopia control therapies (such as myopia control contact lenses) in their practices. Advice on lifestyle modification to reduce myopia risk was only presented to existing myopes, and was generally only discussed if there was time at the end of the eye examination, an issue highlighted by those optometrists working in large multiple ophthalmic practices. If communicating lifestyle advice, clinicians would typically only recommend reducing screen time. Only a minority of participating clinic based optometrists referred progressing myopes to a clinic that does offer myopia control, with some clinicians unsure of who they should refer to *“I’m just referring myopes to their doctor to send them somewhere as I don’t know where to refer them for myopia control”*.

### Perceived barriers to myopia control

Final-year students felt they did not gain enough clinical experience in order to competently practise myopia control. Academic faculty were further concerned that the pressures of target and performance driven clinical practice environments, as well as lack of active management in community-based practices may prevent newly qualified optometrists engaging in myopia control practice once qualified. When probed about integrating additional myopia control clinics into the current undergraduate optometry programme, academic faculty reported the curriculum is at full capacity with one lecturer reporting *“There are no more hours to give. The course is so packed and there is nothing we can cut out.”*

Insufficient education and training was also highlighted as a major barrier to myopia control practice among clinical practice based optometrists, along with a lack of availability of myopia control interventions and limited access to instrumentation such as a corneal topographer or optical biometer. Academic faculty proposed a myopia focused postgraduate programme, along with continuing education and training (CET) and continuing professional development (CPD), to be a viable means by which optometrists can update their knowledge and behaviours and improve clinical performance. Clinic based optometrists reported they had not participated in any myopia focused CET to date, even though they are aware it is available to them, as they felt they needed more skills-based education, such as workshops.

Optometrists in all focus groups were concerned about the financial burdens associated with myopia control, and recognised the significantly shorter test times in large multiples as a barrier to advising on and offering myopia control interventions, stating there was little financial incentive to offering myopia control therapy. Academic optometrists felt the lack of subsidiary funding to Irish optometrists who offer an enhanced optometric service such as myopia control could potentially compromise the profitability of their business. Furthermore, practice based optometrists highlighted that many multiples already limit the number of appointment slots available to children, due to implications on chair time and financial targets.

*“Big multiples typically don’t want us seeing kids, they take up too much chair time.”*

In view of this, academic faculty anticipated that a change in clinical practice will emerge as a response to patient demands for access to certain types of care, leading to myopia control becoming a business priority.

Mobile optometrists or optometrists working in multiples where larger clinical and support teams manage a bigger patient list expressed how the continuity of care and follow up of the same individual over time can be problematic, especially in a clinic where there is variability in optometrists ability to practise myopia control. One practice-based optometrist recounted conducting an aftercare on a myopia control patient previously fitted with multifocal contact lenses, reporting *“I didn’t know how to do the aftercare as I didn’t know anything about myopia control contact lens fitting. I just rebooked them and hoped they were seen by someone who did”* with another optometrist adding *“In multiples we all see each other’s patients, so follow up is difficult.”*

**Figure 1.**
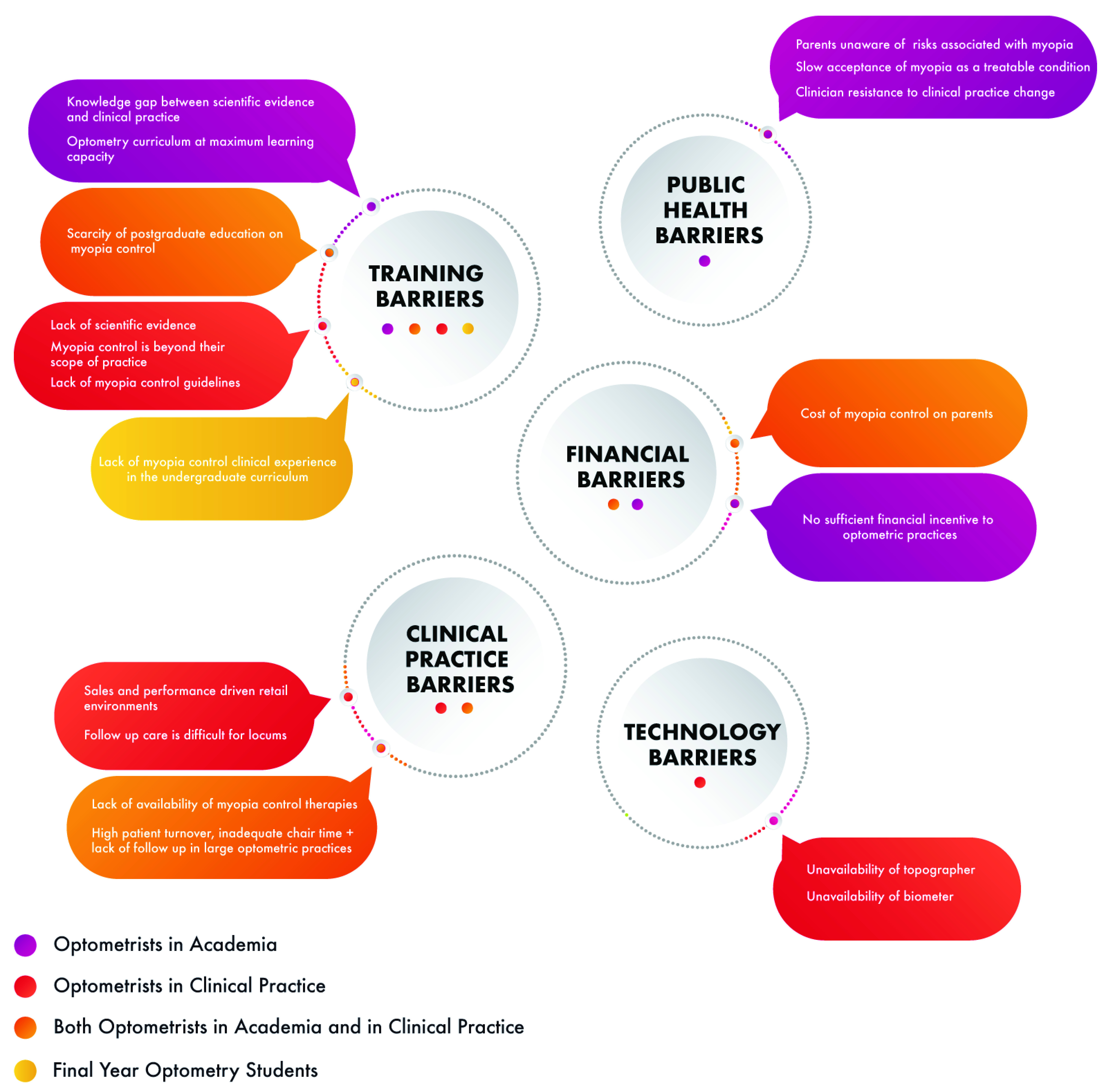
Perceived barriers to the implementation of myopia control interventions, as identified by optometrists in academia and in clinical practice.

Overall 18 different barriers were identified by optometrists in academic and clinical practice settings as well as final year undergraduate students. These were grouped into five specific categories including public-health, clinical practice, technological, financial and training related barriers as outlined in Figure 1.

## Discussion

The key findings to emerge from this study include (i) a disconnect in myopia control knowledge, beliefs and practices between academic optometrists, final year optometry students and clinicians in practice; (ii) the perceived need for extra education, training and guidelines on myopia control and; (iii) the existence of a range of public health issues, clinical practice concerns, financial, technological and other constraints that have limited the translation of myopia control management strategies into routine clinical practice.

Although it is positive to note that students feel myopia control is emphasised in the optometry curriculum, it is concerning that some students have not undertaken any practical form of myopia control, and furthermore may not receive hands-on myopia control experience during their supervised practice placement due to the apparent limited uptake of active myopia management among Irish optometrists. This perceived lack of preparedness is thus likely to limit the provision of emerging myopia control services that are pivotal to tackling the public health consequences of a continued rise in myopia. This is particularly important given that the education and regulatory standards in Ireland are equivalent to that in the UK, with Irish and UK optometrists having a range of permitted competence among the widest in Europe ^35^. However, regardless of their clinical skillset, Optometrists conservative approach to managing myopia is likely underpinned by professional bodies such as the UK College of Optometrists, who have not yet recommended the widespread adoption of myopia control interventions for all myopic children or for those at risk of developing myopia ^22^, despite the release of the International Myopia Institute’s white papers that provide expert and evidence based guidance on the urgent need for new management approaches ^36^. 

The observation that the optometry curricular content is at full capacity with no available teaching hours remaining highlights an important issue. As more evidence based research and optometric management options for myopia are emerging ^37^, reorganisation and innovation in the optometry programme to include myopia control as a practical core competency might better prepare students for contemporary practice and to meet current and future eye care needs. It is important to note optometrists already possess the clinical skills required to carry out myopia control (e.g. multifocal contact lens fitting), therefore a complete reformation of the current curriculum is not required. Within academic faculties, the initiation of “train the trainers” type education would equip clinic supervisors with the knowledge and skills to become fully engaged in myopia control in order to optimise student and patient experience. As the academic programme will influence patterns of clinical practice, the integration of myopia control into optometry programmes is relevant and appropriate, not only in Ireland but internationally given the global nature of the myopia epidemic. Enhancement of ophthalmic training may also assist with the transfer of skills across Europe and other regions ^38^. Measures to ensure a coherent policy to make myopia control competency a compulsory aspect of the European Diploma in Optometry, on which an increasing number of universities are basing their curriculum ^39^, could also be implemented. Optometry education in countries such as the USA, Australia and the UK has responded to the need for therapeutic skills and services in primary care optometry. Myopia management is perhaps even more central to the traditional role of optometry so it is vital that optometry adapts quickly to the ever increasing need for myopia prevention and control. This change needs to be driven by educational providers at undergraduate and postgraduate level.

This study points to a direct need for practically oriented postgraduate training opportunities. Clinic based optometrists identified inadequate and insufficient education and training as well as a lack of availability of myopia control therapies as a fundamental justification for their reluctance to practise myopia control. This supports the results from Wolffsohn *et al.*’s survey that revealed, amongst other barriers, a lack of availability of and information on myopia control means that the majority of international practitioners still prescribe single-vision correction as the primary mode of management for myopic patients, even in countries where the control of myopia progression has become an important clinical goal ^23^. The perceived lack of availability of myopia control management options highlights the need for further education and regulatory guidance. In view of this, the suggestion by participating academics that myopia focused postgraduate education programmes be prioritised seems prudent and would empower clinicians to apply their knowledge and expertise in contact lens fitting, as well as other techniques, into a formal myopia control management routine. However, myopia control focused CET, as well as online myopia management courses and guidelines, have been available to eye care practitioners for a number of years ^40, 41^, yet practitioners still consider themselves too uninformed to expand their scope of practice (which, in Ireland, is defined as one’s ability to practise according to one’s knowledge, skills, competence and experience) ^42^ to include myopia control therapies ^23^. Clinic based optometrists felt skill-based practical learning strategies would instigate the most effective change in professional clinical practice which should be recognised by any emergent CPD programmes. This is consistent with many studies that demonstrate a difference between the proposed versus the actual outcome of continuing education, with a lack of clinical behavioral change associated with the didactic nature of online distance learning ^43, 44^. Correspondingly, interactive and multiple-strategy interventions including regulation have been shown to be highly effective in changing the professional behaviour of clinicians ^44– 46^. Clinician personal attitudes and beliefs will also influence practices ^47^, and although optometrists in our study appeared to have an interest in incorporating myopia control therapies into their clinical practice, study participation may be influenced by their interest in the topic. The study findings, therefore, cannot be interpreted as reflecting the broader profession in Ireland. Other barriers may exist, such as a lack of interest in adopting myopia control strategies, which could be explored across the profession,however our focus groups purposefully allowed optometrists with an interest and/or experience in myopia management to discuss their opinions and contribute in the exploration of this fast evolving area.

This study points to a direct need for practically oriented postgraduate training opportunities. Clinic based optometrists identified inadequate and insufficient education and training as well as a lack of availability of myopia control therapies as a fundamental justification for their reluctance to practise myopia control. This supports the results from Wolffsohn *et al.*’s survey that revealed, amongst other barriers, a lack of availability of and information on myopia control means that the majority of international practitioners still prescribe single-vision correction as the primary mode of management for myopic patients, even in countries where the control of myopia progression has become an important clinical goal ^23^. The perceived lack of availability of myopia control management options highlights the need for further education and regulatory guidance. In view of this, the suggestion by participating academics that myopia focused postgraduate education programmes be prioritised seems prudent and would empower clinicians to apply their knowledge and expertise in contact lens fitting, as well as other techniques, into a formal myopia control management routine. However, myopia control focused CET, as well as online myopia management courses and guidelines, have been available to eye care practitioners for a number of years ^40, 41^, yet practitioners still consider themselves too uninformed to expand their scope of practice (which, in Ireland, is defined as one’s ability to practise according to one’s knowledge, skills, competence and experience) ^42^ to include myopia control therapies ^23^. Clinic based optometrists felt skill-based practical learning strategies would instigate the most effective change in professional clinical practice which should be recognised by any emergent CPD programmes. This is consistent with many studies that demonstrate a difference between the proposed versus the actual outcome of continuing education, with a lack of clinical behavioral change associated with the didactic nature of online distance learning ^43, 44^. Correspondingly, interactive and multiple-strategy interventions including regulation have been shown to be highly effectivein changing the professional behaviour of clinicians44–46. Clinician personal attitudes and beliefs will also influence practices ^47^, and although optometrists in our study appeared to have an interest in incorporating myopia control therapies into their clinicalpractice, study participation may be influenced by their interest in the topic. The study findings, therefore, cannot be interpreted as reflecting the broader profession in Ireland. Other barriersmay exist, such as a lack of interest in adopting myopia control strategies, which could be explored across the profession,however our focus groups purposefully allowed optometrists with an interest and/or experience in myopia management to discuss their opinions and contribute in the exploration of this fastevolving area.

The observation by clinic based participants that there is a lack of substantial evidence to advocate outdoor activity is of particular concern. Interventions to increase time spent outdoors are well documented and have proven to be effective in terms of reducing the risk of myopia development ^9^, with the additional benefits of minimal cost involved, low possibility of adverse effects and other positive health outcomes. The provision of advice on myopia prevention strategies is subjective and depends heavily on the knowledge and attitude of the optometrist toward myopia and its control. As frontline providers of eyecare, optometrists have a responsibility to educate at-risk patients on prophylactic measures to prevent the onset of myopia in the first instance, in addition to communicating lifestyle advice and discussing possible interventions, including their limitations, to existing myopes and parents.

The financial barriers identified corroborate and extend previous findings that highlight clinicians are concerned that myopia control is expensive, timely, and occupies valuable chair time ^23^. The academic faculty’s suggestion that increased demand for myopia control would provoke a change in clinical practice is aligned with a motive previously described by Lomas ^48^, who recognised that patients represent a group of consumers who now enquire about treatment options, and have more choice than ever in selecting an optometrist or an optometric practice ^49– 51^. Health care consumerism thus advocates patients’ involvement in their own healthcare decisions ^49^, and is identified as a central requirement in health system reform ^52, 53^. In the same manner, increased demand for myopia control treatment in a competitive market should motivate practitioners to address their perceived barriers to myopia control and offer a range of services to meet patient needs.

Academic faculty further recognised that demand for myopia control therapy will depend on patient and parental education on myopia and its control. This is an important aspect that needs to be addressed through appropriate public health policy given that parents have limited understanding of the causes and risk factors associated with myopia ^54^. Consequently, any strategy aiming to control myopia progression must target parents as well as practitioners, in order to address the myopia knowledge gap that currently exists amongst all stakeholders.

### Limitations

A known disadvantage of any focus group discussion is the possibility that participants may not express their honest and personal opinions about the topic. However, as focus groups can yield deeper insights into behaviour and motivations we felt this was opportune for an emerging clinical practice. Furthermore, in a rapidly evolving field of research, focus groups provide an efficient and timely contribution of data.

The focus group moderator was an academic optometrist whose knowledge of myopia control was fundamental in order to conduct effective follow-up and constructively frame participants' responses with the discussions. However, pre-specified questions were used to prevent the moderator’s personal biases influencing participants' exchange of ideas and thereby generate data based on the interactions within the group discussion.

## Conclusion

Although these focus groups cannot be assumed to be entirely reflective of clinical optometry practice, our findings demonstrate a marked lack of willingness to engage in the practice of myopia control among final year students and clinical optometrists. Education, training, finance, and time restrictions, as well as limited availability of myopia control therapies were among the main barriers identified by participants. In order to develop a coherent profession-wide response to myopia, our findings indicate a need for increased clinical myopia control practical experience within the undergraduate program as well as enhanced postgraduate education that compliments the available online training through a hands-on practical approach. As myopia is a global epidemic and optometrists are the primary eyecare profession tasked with addressing myopia in many countries, the importance of the role of optometrists in advocating for and providing myopia control is relevant not only in Ireland, but internationally.

## Data availability

### Underlying data

Full de-identified transcripts available upon request from the corresponding author, SMC (saoirse.mccrann@tudublin.ie). The data are not publicly available due to their containing information that could compromise the privacy of research participants. Access will be granted to researchers that are planning similar future studies.

### Extended data

Figshare: Pre specified questions; Is optometry ready for myopia control? Education and other barriers to the treatment of myopia. https://doi.org/10.6084/m9.figshare.10260347.v1 ^30^.

### Reporting guidelines

Figshare: COREQ checklist for ‘Is optometry ready for myopia control? Education and other barriers to the treatment of myopia’. https://doi.org/10.6084/m9.figshare.10266638.v1 ^39^.

Extended data and completed reorting guidelines are available under the terms of the Creative Commons Attribution 4.0 International license (CC-BY 4.0).

## References

[1] Holden BA Fricke TR Wilson DA: Global Prevalence of Myopia and High Myopia and Temporal Trends from 2000 through 2050. *Ophthalmology.* 2016;123(5):1036–1042. 10.1016/j.ophtha.2016.01.006 26875007

[2] Pan CW Ramamurthy D Saw SM: Worldwide prevalence and risk factors for myopia. *Ophthalmic Physiol Opt.* 2012;32(1):3–16. 10.1111/j.1475-1313.2011.00884.x 22150586

[3] Lin LL Shih YF Hsiao CK: Prevalence of myopia in Taiwanese schoolchildren: 1983 to 2000. *Ann Acad Med Singapore.* 2004;33(1):27–33. 15008558

[4] Foster PJ Jiang Y: Epidemiology of myopia. *Eye (Lond).* 2014;28(2):202–208. 10.1038/eye.2013.280 24406412 PMC3930282

[5] Saw SM Gazzard G Shih-Yen EC: Myopia and associated pathological complications. *Ophthalmic Physiol Opt.* 2005;25(5):381–391. 10.1111/j.1475-1313.2005.00298.x 16101943

[6] Tideman JW Snabel MC Tedja MS: Association of Axial Length With Risk of Uncorrectable Visual Impairment for Europeans With Myopia. *JAMA Ophthalmol.* 2016;134(12):1355–1363. 10.1001/jamaophthalmol.2016.4009 27768171

[7] Pararajasegaram R: VISION 2020-the right to sight: from strategies to action. *Am J Ophthalmol.* 1999;128(3):359–360. 10.1016/s0002-9394(99)00251-2 10511033

[8] Morgan RW Speakman JS Grimshaw SE: Inuit myopia: an environmentally induced "epidemic"? *Can Med Assoc J.* 1975;112(5):575–577. 1116086 PMC1956268

[9] Xiong S Sankaridurg P Naduvilath T: Time spent in outdoor activities in relation to myopia prevention and control: a meta-analysis and systematic review. *Acta Ophthalmol.* 2017;95(6):551–566. 10.1111/aos.13403 28251836 PMC5599950

[10] Mountjoy E Davies NM Plotnikov D: Education and myopia: assessing the direction of causality by mendelian randomisation. *BMJ.* 2018;361:k2022. 10.1136/bmj.k2022 29875094 PMC5987847

[11] Huang HM Chang DS Wu PC: The Association between Near Work Activities and Myopia in Children-A Systematic Review and Meta-Analysis. *PLoS One.* 2015;10(10):e0140419. 10.1371/journal.pone.0140419 26485393 PMC4618477

[12] Huang J Wen D Wang Q: Efficacy Comparison of 16 Interventions for Myopia Control in Children: A Network Meta-analysis. *Ophthalmology.* 2016;123(4):697–708. 10.1016/j.ophtha.2015.11.010 26826749

[13] Wildsoet CF Chia A Cho P: IMI - Interventions Myopia Institute: Interventions for Controlling Myopia Onset and Progression Report. *Investig Opthalmology Vis Sci.* 2019;60(3):M106–M131. 10.1167/iovs.18-25958 30817829

[14] Lam CSY Tang WC Tse DY: Defocus Incorporated Multiple Segments (DIMS) spectacle lenses slow myopia progression: a 2-year randomised clinical trial. *Br J Ophthalmol.* 2020;104(3):363–368. 10.1136/bjophthalmol-2018-313739 31142465 PMC7041503

[15] Goss DA Grosvenor T: Rates of childhood myopia progression with bifocals as a function of nearpoint phoria: consistency of three studies. *Optom Vis Sci.* 1990;67(8):637–640. 10.1097/00006324-199008000-00015 2216333

[16] Cheng D Schmid KL Woo GC: Randomized trial of effect of bifocal and prismatic bifocal spectacles on myopic progression: two-year results. *Arch Ophthalmol.* 2010;128(1):12–9. 10.1001/archophthalmol.2009.332 20065211

[17] Hasebe S Ohtsuki H Nonaka T: Effect of progressive addition lenses on myopia progression in Japanese children: a prospective, randomized, double-masked, crossover trial. *Investig Opthalmology Vis Sci.* 2008;49(7):2781–9. 10.1167/iovs.07-0385 18579755

[18] Wu PC Chuang MN Choi J: Update in myopia and treatment strategy of atropine use in myopia control. *Eye (Lond).* 2019;33(1):3–13. 10.1038/s41433-018-0139-7 29891900 PMC6328548

[19] McCrann S Flitcroft I Strang NC: Myopia Outcome Study of Atropine in Children (MOSAIC): an investigator-led, double-masked, placebo-controlled, randomised clinical trial protocol [version 1; peer review: 2 approved, 1 approved with reservations]. *HRB Open Res.* 2019;2:15. 10.12688/hrbopenres.12914.1 32002514 PMC6973533

[20] Azuara-blanco A Logan N Strang N: Low-dose (0.01%) atropine eye-drops to reduce progression of myopia in children: a multicentre placebo-controlled randomised trial in the UK (CHAMP-UK)—study protocol. *Br J Ophthalmol.* 2019; pii: bjophthalmol-2019-314819. 10.1136/bjophthalmol-2019-314819 31653669

[21] Jones L Drobe B González-Méijome JM: IMI – Industry Guidelines and Ethical Considerations for Myopia Control Report. *Investig Opthalmology Vis Sci.* 2019;60(3):M161–M183. 10.1167/iovs.18-25963 30817831

[22] The College of Optometrists: Guidance for Optometrists- Myopia Management.2019 Reference Source

[23] Wolffsohn JS Calossi A Cho P: Global trends in myopia management attitudes and strategies in clinical practice. *Cont Lens Anterior Eye.* 2016;39(2):106–116. 10.1016/j.clae.2016.02.005 26895778

[24] Zloto O Wygnanski-Jaffe T Farzavandi SK: Current trends among pediatric ophthalmologists to decrease myopia progression-an international perspective. *Graefes Arch Clin Exp Ophthalmol.* 2018;256(12):2457–2466. 10.1007/s00417-018-4078-6 30074069

[25] Jung JJ Lim EH Baek SH: Attempts to reduce the progression of myopia and spectacle prescriptions during childhood: a survey of eye specialists. *Korean J Ophthalmol.* 2011;25(6):417–20. 10.3341/kjo.2011.25.6.417 22131779 PMC3223709

[26] Douglass A Keller PR He M: Knowledge, perspectives and clinical practices of Australian optometrists in relation to childhood myopia. *Clin Exp Optom.* 2020;103(2):155–166. 10.1111/cxo.12936 31257703

[27] Healio: Round table: Pediatric ophthalmologists address ‘hot topic’ of stemming myopia progression.Ocular Surgery News U.S. Edition. Accessed July 20, 2017.2016 Reference Source

[28] NEJM Catalyst: What Is Risk Management in Healthcare? *Catal Carryover.* 2018;4(2). Reference Source

[29] Bowen GA: Naturalistic inquiry and the saturation concept: a research note. *Qual Res.* 2008;8(1):137–152. 10.1177/1468794107085301

[30] McCrann S: Pre specified questions; Is optometry ready for myopia control? Education and other barriers to the treatment of myopia. *Figshare J Contrib.* 2019 10.6084/m9.figshare.10260347.v1 PMC7542256 33083690

[31] Boyatzis RE: Transforming Qualitative Information: Thematic Analysis and Code Development.Sage Publications.1998 Reference Source

[32] McCrann S Flitcroft I Loughman J: Repository: COREQ checklist for Is Optometry Ready for Myopia Control? Education and other Barriers to the Treatment of Myopia. *ARROW @ TU Dublin.* 2019;349–357. 10.21427/5474-rd62 PMC7542256 33083690

[33] Ott DeKinder J: Optometry Times: Fit multifocal lenses for older and younger patients | Optometry Times.Accessed February 16, 2019.2017 Reference Source

[34] Coopervision: Fitting MiSight ^TM^ to Children.Accessed February 16, 2019. Reference Source

[35] Association of Optometrists: Europe and optometry. Accessed April 25, 2019. Reference Source

[36] Wolffsohn JS Flitcroft DI Gifford KL: IMI – Myopia Control Reports Overview and Introduction. *Investig Opthalmology Vis Sci.* 2019;60(3):M1–M19. 10.1167/iovs.18-25980 30817825 PMC6735780

[37] Flanagan SC Saunders KJ: Optometry in Practice The Optometric Management of Childhood Myopia: A Review of the Evidence.2019 Reference Source

[38] The College of Optometrists: EU or EEA nationals.Accessed June 24, 2019. Reference Source

[39] The European Council of Optometry and Optics: Pilot Accreditation Process For Exemption from the Examinations and Portfolio of The European Diploma in Optometry.2012; Accessed April 25, 2019. Reference Source

[40] Allen P Radhakrishnan H: Continuing education CET, Myopia. *Optician.* 2010;18–24. Accessed January 8, 2019. Reference Source

[41] Brien Holden Vision Institute: Guidelines for Myopia Management.Accessed January 9, 2019. Reference Source

[42] Optical Registration Board: Code of Professional Conduct and Ethics for Optometrists.2015; Accessed January 30, 2019. Reference Source

[43] Davis D O’Brien MA Freemantle N: Impact of formal continuing medical education: do conferences, workshops, rounds, and other traditional continuing education activities change physician behavior or health care outcomes? *JAMA.* 1999;282(9):867–74. 10.1001/jama.282.9.867 10478694

[44] Cantillon P Jones R: Does continuing medical education in general practice make a difference? *BMJ.* 1999;318(7193):1276–1279. 10.1136/bmj.318.7193.1276 10231265 PMC1115655

[45] Norman GR Van der Vleuten C Newble D: International Handbook of Research in Medical Education. Springer.2002 10.1007/978-94-010-0462-6

[46] Robertson R Jochelson K: Interventions That Change Clinician Behaviour: Mapping the Literature.2006 Reference Source

[47] Laws RA Kemp LA Harris MF: An exploration of how clinician attitudes and beliefs influence the implementation of lifestyle risk factor management in primary healthcare: A grounded theory study. *Implement Sci.* 2009;4(1):66. 10.1186/1748-5908-4-66 19825189 PMC2770564

[48] Lomas J: Teaching Old (and Not so Old) Docs New Tricks: Effective Ways to Implement Research Findings. In: Dunn EV, Norton PV, Stewart M, Eds. *Disseminating Research, Changing Practice* Newbury Park: Sage.1994 Reference Source

[49] Sivakumar A: Professional management for eye care. *Community Eye Health.* 2006;19(59):50–51. 17491720 PMC1705624

[50] The Competition Authority: Competition in Professional Services. *Optometrists.* 2006; Accessed January 10, 2019. Reference Source

[51] Smith J Ham C Parker H: To Market, to Market: What Future for Primary Care?Executive Summary.2005; Accessed January 10, 2019. Reference Source

[52] Segal L: The importance of patient empowerment in health system reform. *Health Policy.* 1998;44(1):31–44. 10.1016/s0168-8510(98)00007-4 10180200

[53] Coulter A: Managing demand at the interface between primary and secondary care. *BMJ.* 1998;316(7149):1974–1976. 10.1136/bmj.316.7149.1974 9641944 PMC1113413

[54] McCrann S Flitcroft I Lalor K: Parental attitudes to myopia: a key agent of change for myopia control? *Ophthalmic Physiol Opt.* 2018;38(3):298–308. 10.1111/opo.12455 29691921

