# Supplementary material for: Is optometry ready for myopia control? Education and other barriers to the treatment of myopia
Source: HRB Open Res. 2020 Apr 23;2:30. Originally published 2019 Nov 15. [Version 2] doi: 10.12688/hrbopenres.12954.2 (PMC7542256; doi:10.12688/hrbopenres.12954.2)
Supplement: Peer review history

# Peer review: Reviewer response for version 2

**DOI:** 10.21956/hrbopenres.14135.r27858

**Published:** 2020-10-07

**Authors:** Pauline Kang

**Affiliations:** 1 School of Optometry and Vision Science, The University of New South Wales, Sydney, New South Wales, Australia

This study investigated perceived barriers to implementation of myopia control in optometric clinical practice in Ireland. Although there is a growing body of evidence of effective interventions for controlling progressive myopia in children, there is a clear disconnect with optometrists not adopting evidence-based myopia management in clinical practice. This is a very well written manuscript, and I have minor comments to add from the previous reviewer.

 Abstract background: please state that this study was aimed to understand attitudes of optometrists/educators/students in Ireland.

 Abstract conclusion: lack of availabilities of therapies is specific to optometrists in Ireland, so I would include this as it currently sounds like this is applicable to all optometrists which is not true.

 Abstract results and main text methods: were academic participants actively practicing optometrists?

 Main text results: very little discussion of therapeutic interventions. Are optometrists in the UK/Ireland allowed to prescribe therapeutic drugs? There was mention of referral to optometrists who prescribe myopia management. Any discussion on co-management with ophthalmologists if optometrists cannot prescribe atropine?

 Some students feel reluctant to prescribe myopia management during their training as they have limited exposure to paediatric patients. Was there any mention of this as a potential barrier?

 Limitation: I think it will be worthwhile to include that the academic faculty and students were from one institution. Students from other institutions may have different perceptions so this is a limited focus group.

Is the work clearly and accurately presented and does it cite the current literature?

Yes

If applicable, is the statistical analysis and its interpretation appropriate?

Not applicable

Are all the source data underlying the results available to ensure full reproducibility?

Yes

Is the study design appropriate and is the work technically sound?

Yes

Are the conclusions drawn adequately supported by the results?

Yes

Are sufficient details of methods and analysis provided to allow replication by others?

Yes

Reviewer Expertise:

Myopia, myopia control, orthokeratology, atropine, optometry.

I confirm that I have read this submission and believe that I have an appropriate level of expertise to confirm that it is of an acceptable scientific standard.

# Peer review: Reviewer response for version 2

**DOI:** 10.21956/hrbopenres.14135.r27727

**Published:** 2020-09-15

**Authors:** Xiangui He, Hui Xie

**Affiliations:** 1 Department of Preventative Ophthalmology, Shanghai Eye Disease Prevention and Treatment Center, Shanghai, China; 2 Shanghai Eye Disease Prevention and Treatment Center, Shanghai, China

The authors designed a qualitative study to explore the education and other barriers to the treatment of myopia. This study provided interesting information, but some elements of this manuscript need further explanation and improvement. My comments are as follows:

**Introduction:**

- Last paragraph: “This prospective study was……”. By stating a prospective design for a study, this means that there is a follow-up procedure to collect time-varying information from the participants. However, the follow-up procedure was not seen in this manuscript and possibly not suitable for a qualitative study.

 

**Methods:**

- Last paragraph: the authors stated they collected basic information (practicing duration, job title, etc.) from clinic-based optometrist participants, however, the authors did not clarify what information was collected from the final-year student participants and the faculty participants.
  This possibly impeded the presentation of sample characteristics in the results part of the manuscript.

 

**Results:**

- First paragraph: could you provide some summary statistics for describing the sample characteristics?  Recommend using a table to present the basic information.
- The authors stated they employed pre-specified codebook or questions to guide the FGD. Were these pre-specified topics applied to guide the data analysis (induction)? Were there any new themes emerging during data analysis? From the results, it seemed that there was no new theme emerging during data analysis.
- From the results part, it seemed that it was difficult to get the core content of the analysis result. The authors did not present clearly the themes being summarized. The induction and summarization seemed to be inadequate. For example:
  For the theme “Knowledge and attitudes toward myopia and myopia control”, recommend first placing the subtheme(1) eagerness to learn about how to incorporate myopia control into clinical practice; and followingly placing the corresponding quotes that are the direct comments from the participants; (2) perceived benefit from a set of recommended guidelines and workshops on myopia control; and followingly quoting the subtheme with participants’ comments. The same summarization and presentation for other themes.
- It is also recommended that the difference in one theme/subtheme among different types of participants (clinic-based optometrist participants, student participants, and faculty participants) should be summarized and be presented with corresponding quotes in a systematic and clear manner.
- The quotes should be marked with corresponding participants’ labels or codes. e.g.
   “xxxxxxxxx”—P01.
- The themes/subthemes should be quoted by multiple comments from the participants, but not solely one single comment.

 

**Discussion:**

- Two paragraphs were overlapped. Check it out.

**General:**

- It was possibly better to use concise sentences to make the text more readable.

Is the work clearly and accurately presented and does it cite the current literature?

Partly

If applicable, is the statistical analysis and its interpretation appropriate?

Not applicable

Are all the source data underlying the results available to ensure full reproducibility?

Partly

Is the study design appropriate and is the work technically sound?

Partly

Are the conclusions drawn adequately supported by the results?

Yes

Are sufficient details of methods and analysis provided to allow replication by others?

No

Reviewer Expertise:

NA

We confirm that we have read this submission and believe that we have an appropriate level of expertise to confirm that it is of an acceptable scientific standard, however we have significant reservations, as outlined above.

# Peer review: Reviewer response for version 2

**DOI:** 10.21956/hrbopenres.14135.r27343

**Published:** 2020-05-29

**Authors:** Julie-Anne Little

**Affiliations:** 1 Centre for Optometry and Vision Science Research, Biomedical Sciences Research Institute, Ulster University, Coleraine, UK

Thanks to the authors for their revisions to the manuscript, which have addressed many of my concerns. There are some remaining points that would be helpful to address:

1. I still do not think your description of study design (‘This prospective study was designed to elucidate the current knowledge gaps and attitudes of optometrists in Ireland toward myopia control’) captures what you did in this study - and doesn’t reflect that you asked optometric educators nor future optometrists about myopia management.
  I suggest changing this to: ‘This prospective study was designed to elucidate the current knowledge gaps and attitudes of optometric educators, optometry students and clinical optometrists in Ireland toward myopia control’. 
2. While the authors have added more information in the introduction regarding what potential myopia control options are available in Ireland, this is still not mentioned as a barrier in the discussion. The availability of myopia control therapies/lenses is surely a key barrier limiting how myopia management practice can become more established. This should be included in the discussion. For example, the author could outline what progress is being made in updating prescribing regulations so that low dose atropine is available to prescribe. 
3. In the discussion, financial barriers are perceived which ‘highlight clinicians are concerned that myopia control is expensive, timely, and occupies valuable chair time’. 
  To understand the context of this, it would be useful to reflect that this is perceived as a barrier as there is not a tradition of charging for ophthalmic services in primary eye beyond routine sight testing and thus for myopia control to be implemented, funding models which reflect the multiple appointments required and time taken with patients would need to be developed.
4. Finally, given the current status and the limitations of myopia management becoming more established in clinical practice, the first line of Abstract Conclusion reads rather negatively towards optometrists. Please re-evaluate.

Is the work clearly and accurately presented and does it cite the current literature?

Yes

If applicable, is the statistical analysis and its interpretation appropriate?

Not applicable

Are all the source data underlying the results available to ensure full reproducibility?

No

Is the study design appropriate and is the work technically sound?

Partly

Are the conclusions drawn adequately supported by the results?

Partly

Are sufficient details of methods and analysis provided to allow replication by others?

No

Reviewer Expertise:

Optometry, paediatric vision, cataract, developmental disability.

I confirm that I have read this submission and believe that I have an appropriate level of expertise to confirm that it is of an acceptable scientific standard, however I have significant reservations, as outlined above.

# Peer review: Reviewer response for version 1

**DOI:** 10.21956/hrbopenres.14036.r26928

**Published:** 2019-12-09

**Authors:** Julie-Anne Little

**Affiliations:** 1 Centre for Optometry and Vision Science Research, Biomedical Sciences Research Institute, Ulster University, Coleraine, UK

**Introduction**:

 Myopia is a refractive error – describing it as a ‘visual disorder’ makes it sound like a disease. 

 Can you be more specific at the end of second paragraph: ‘appear to’ sounds vague with regard to prolonged near work and it would be useful to elucidate between differences in what could drive myopia development compared to progression, as you have described with time outdoors.

 What about myopia control progressive addition spectacle lenses? They are not mentioned as an approach, and while I know that their benefit in myopia retardation is less effective than antimuscarinic agents and contact lenses, it is worth mentioning, and it would then make more sense why you refer to ‘single vision’ spectacles later as the conventional approach.

 The authors need to describe more fully what ‘slow myopia progression’ with myopia control is successful looks like, i.e. how much myopia could be avoided?

 Myopia control is still an emerging practice, and is not a treatment that is yet offered by the NHS in the UK. The authors should acknowledge this, and cite the guidance produced by Professional bodies (i.e. the College of Optometrists, the Association of Optometrists). I know this is a rapidly changing area, and that aforementioned professional guidance is possibly a little conservative (even since it was written 2-3 years ago), but to me it is concerning that an academic optometrist in the focus group stated that a person would be negligent if not offering myopia control, and yet current regulatory standards for optometrists would not reflect that. The authors need to provide further context as to what extent myopia control is practiced by optometrists in Ireland. 

 What does “limit the transition to a risk-focussed model” at end of introduction mean?

**Methods**:

 How did recruitment of community optometrists occur? How did you mitigate the bias in this sample? i.e. that they participated in the study because they were interested in and positive about myopia control. This probable bias is highlighted in the fact that a major theme that emerges was an interest in myopia control therapies in clinical practice. I do not think your sample was representative of the optometric profession in Ireland, and thus a weakness in answering your study aim.

 I assume you had a first focus group with n=6 and the second occurred at a later date?

 What was the protocol for the focus group discussion? How was discussion initiated and prompted? How was the effect of the researcher being perceived as an academic optometrist controlled for in the focus groups with a) students (who may then have been reluctant to admit their lack of understanding of the topic to their lecturer) and b) community optometrists (who may have been intimidated to admit their lack of knowledge to a fellow professional)?

**Results**:

 ‘Practice-based’ and ‘clinic based’ is used in the results, but community optometrists used earlier. Be consistent in naming optometric groups.

 Focus group were relatively new to professionals of 1-11 year range: again, does this limit drawing conclusions about myopia management to the profession as a whole?

 Surely a barrier that should be discussed is the availability of myopia control therapies/lenses. In the introduction, the authors note that there are numerous available therapies – but then availability is highlighted as a clinical practice barrier in the Figure. However, this is not discussed in the text. Are optometrists unaware of what to order? Are they not available/distributed in Ireland?

 Overall I am not persuaded that the authors have answered their research question with the methodology employed. They sought to “elucidate the current practice, knowledge gaps and attitudes of optometrists in Ireland toward myopia control, and thereby identify perceived barriers that may limit the transition to a risk-focussed model of myopia management in primary care practice”.

 The lack of information in the way in which community optometrists in Ireland were recruited (and the small sample size) means that this work does not probably capture the current practice and knowledge gaps for the optometry profession in Ireland and likely is a biased sample. This is underlined in the relatively small number of years of experience of the focus group members, and the clear bias of interest in myopia control suggests that these optometrists are not necessarily typical of the optometric workforce.

 There is interesting work here, but the authors need to make significant revision to their manuscript, fully describing their recruitment and focus group methodology and acknowledge the limitation of their study design to address their research question.

Is the work clearly and accurately presented and does it cite the current literature?

Yes

If applicable, is the statistical analysis and its interpretation appropriate?

Not applicable

Are all the source data underlying the results available to ensure full reproducibility?

No

Is the study design appropriate and is the work technically sound?

Partly

Are the conclusions drawn adequately supported by the results?

Partly

Are sufficient details of methods and analysis provided to allow replication by others?

No

Reviewer Expertise:

Optometry, paediatric vision, cataract, developmental disability.

I confirm that I have read this submission and believe that I have an appropriate level of expertise to state that I do not consider it to be of an acceptable scientific standard, for reasons outlined above.

# Response

**Published:** 2020-04-14

**Authors:** Saoirse McCrann

**Affiliations:** Technological University Dublin, Ireland

Author Response 23 Sep 2019

**Saoirse McCrann, **Technological University Dublin, Ireland

**Reviewer 1: Julie-Anne Little**

  

**Reviewer comment 1**

Myopia is a refractive error – describing it as a ‘visual disorder’ makes it sound like a disease. 

**Author response 1**

Myopia is the most frequent cause of distance impairment in the world and therefore the authors would argue that myopia is more than just a refractive error, but a condition characterised by a pattern of abnormal ocular growth. ^1^ As the vast majority of eye care clinicians still prescribe single vision interventions to young myopes, ^2^ and as there is a lack of recognition of the health risks for eye disease associated with myopia in Ireland, ^3^ ﻿we believe it is important myopia is recognised as more than just an optical error. We have updated our introduction in the Revised Manuscript to read:

*As myopia is a condition characterised by a pattern of abnormal ocular growth and high myopia is a leading cause of irreversible vision impairment and blindness, the increasing levels of myopia arguably represents one of the most important ophthalmic public health threats of our time*

**Reviewer comment 2**

Can you be more specific at the end of second paragraph: ‘appear to’ sounds vague with regard to prolonged near work and it would be useful to elucidate between differences in what could drive myopia development compared to progression, as you have described with time outdoors.

**Author response 2**

Thank you for this observation. This sentence in the introduction has been updated to read

*Other factors, such as more time spent in education,* ^4^ *and prolonged or continuous near work,* ^5^ *have been associated with an increased risk of myopia development and progression.*

**Reviewer comment 3**

What about myopia control progressive addition spectacle lenses? They are not mentioned as an approach, and while I know that their benefit in myopia retardation is less effective than antimuscarinic agents and contact lenses, it is worth mentioning, and it would then make more sense why you refer to ‘single vision’ spectacles later as the conventional approach.

**Author response 3**

Thank you for this observation. We did not include bifocals/progressive addition lenses as only myopes with near esophoria or an accommodative lag have a statistically better response to this treatment, which should not warrant a change in clinical practice. ^6^ However, as it helps clarify our reference to single vision spectacles we have updated the sentence and also included defocus modifying spectacle lenses (DIMS)

*A range of optical and pharmacological interventions, such as atropine eye drops (at varying concentrations), multifocal contact lenses, orthokeratology and defocus modifying spectacle lenses (DIMS) have been demonstrated to reduce myopia progression when compared with single vision spectacle lenses or placebo. ^7–9^ Studies have also indicated bifocal and progressive addition spectacle lenses can also slow the progression of myopia in children with near-point esophoria and larger accommodative lags ^10–12^*

**Reviewer comment 4**

The authors need to describe more fully what ‘slow myopia progression’ with myopia control is successful looks like, i.e. how much myopia could be avoided?

**Author response 4**

Thank you for this comment. Although difficult to predict how much myopia can be avoided in an individual due to a multitude of variable risk factors including age, parental myopia or environmental influences, we have updated the Manuscript in line with the 2019 IMI reports which detail the effectiveness of different myopia control

﻿treatment strategies. ^8^The updated sentence reads as follows

*A number of contact lens options for managing myopia are also commercially available in Europe and are reported to slow myopia progression by approximately 30% to 60%.* ^8^ *.*

**Reviewer comment 5**

Myopia control is still an emerging practice, and is not a treatment that is yet offered by the NHS in the UK. The authors should acknowledge this, and cite the guidance produced by Professional bodies (i.e. the College of Optometrists, the Association of Optometrists). I know this is a rapidly changing area, and that aforementioned professional guidance is possibly a little conservative (even since it was written 2-3 years ago), but to me it is concerning that an academic optometrist in the focus group stated that a person would be negligent if not offering myopia control, and yet current regulatory standards for optometrists would not reflect that. The authors need to provide further context as to what extent myopia control is practiced by optometrists in Ireland. 

**Author response 5**

Thank you for this comment. This was the first ever study that aimed to understand myopia control practices in Ireland, which was previously unknown. Therefore

further context as to what extent myopia control was practiced at the time of data collection is not available. We do however acknowledge that myopia control is an emerging practice

*As more evidence based research and optometric management options for myopia are emerging,* ^13^ 

And provide more detail on myopia management options available to Optometrists in Ireland

*Low dose atropine is not readily available as a licensed product in Europe but is accessible through clinical trials, with the recruitment of trial participants dependent on Optometrist referrals.* ^14–16^ *A number of contact lens options for managing myopia are also commercially available in Europe and are reported to slow myopia progression by approximately 30% to 60%.* ^8^ *. These include multifocal contact lenses, frequently fitted ‘off- label’,* ^17^ *novel myopia control lenses (becoming more readily available in Ireland and the UK),* ^18^ *and orthokeratology contact lenses.*

Furthermore, the updated Manuscript now makes reference to the UK College of Optometrist Report and the IMI white papers as follows 

*Optometrists conservative approach to managing myopia is likely underpinned by professional bodies such as the UK College of Optometrists, who have not yet recommended the widespread adoption of myopia control interventions for all myopic children or for those at risk of developing myopia,* ^18^ *despite the release of the International Myopia Institute’s white papers that provide expert and evidence based guidance on the urgent need for new management approaches.* ^19^

**Reviewer comment 6**

What does “limit the transition to a risk-focussed model” at end of introduction mean?

**Author response 6**

Risk focussed management is clinically detecting, monitoring, assessing, mitigating, and preventing risks. ^20^ In terms of myopia control, a risk-focused model can be interpreted as the approach taken by eye care professionals to detect myopia, reduce myopia progression and prevent the risk of associated eye conditions. The aim of our study is to identify perceived barriers to adopting such a risk focused management model. We have updated our Manuscript and references as follows

*Thereby identify perceived barriers that may limit the transition to a risk-focussed management model of myopia in primary care practice, wherein eye care professionals would implement clinical management strategies designed to limit the risk of myopia onset, progression and development of future myopia-related complications for individual patients .* ^20^

**Reviewer comment 7**

Methods:

How did recruitment of community optometrists occur?

**Author response 7**

Thank you for this comment. Further details on recruitment is now reported in the methods as follows

*An ‘invitation to participate’ email request was sent to Optometric practices for administrators to disseminate amongst their clinic based optometrists. Final year optometry students and optometry faculty at Technological University Dublin (TU Dublin) were﻿ ﻿contacted directly through the study investigator via email and invited to participate in the study*

**Reviewer comment 8**

How did you mitigate the bias in this sample? i.e. that they participated in the study because they were interested in and positive about myopia control. This probable bias is highlighted in the fact that a major theme that emerges was an interest in myopia control therapies in clinical practice. I do not think your sample was representative of the optometric profession in Ireland, and thus a weakness in answering your study aim.

**Author response 8**

Focus groups are not intended to statistically represent a broader population, but instead aim to gain an in-depth understanding of an idea or issue from a selected group of individuals. ^21^ Optometrists may have participated in the study because they were interested in and positive about myopia control, but the issues raised by these participants are important as it is likely that other Optometrists may face the same issues. We do, however, recognise that the focus groups are not designed to quantify Ireland’s Optometry practices and have updated the discussion of our Manuscript as follows

*Although optometrists in our study appeared to have an interest in incorporating myopia control therapies into their clinical practice, study participation may be influenced by their interest in the topic. The study findings, therefore, cannot be interpreted as reflecting the broader profession in Ireland. Other barriers may exist, such as a lack of interest in adopting myopia control strategies, which could be explored across the profession, however our focus groups purposefully allowed optometrists with an interest and/or experience in myopia management to discuss their opinions and contribute in the exploration of this fast evolving area.*

This is also highlighted in our conclusion as follows

*Although these focus groups cannot be assumed to be entirely reflective of clinical optometry practice*

**Reviewer comment 9**

I assume you had a first focus group with n=6 and the second occurred at a later date?

**Author response 9**

Yes, two separate focus groups were carried out. This is now clarified in the Manuscript as follows

*Two separate focus group discussions involved optometrists (n=6 in each) working in optometry practices across Ireland*

**Reviewer comment 10**

What was the protocol for the focus group discussion? How was discussion initiated and prompted? How was the effect of the researcher being perceived as an academic optometrist controlled for in the focus groups with a) students (who may then have been reluctant to admit their lack of understanding of the topic to their lecturer) and b) community optometrists (who may have been intimidated to admit their lack of knowledge to a fellow professional)?

**Author response 10**

The focus group was initiated by informing participants of the nature of the study prior to obtaining verbal informed consent using audio recording. The discussion was prompted by pre-specified questions informed by a review of the literature (see extended data). The pre-specified questions were also used to prevent the moderator’s personal biases influencing participants' exchange of ideas and therefore generate data based on interactions within the group discussion.

A known disadvantage of any focus group discussion is the possibility that participants may not express their honest and personal opinions about the topic. However, as focus groups can yield deeper insights into behaviour and motivations, we felt this was opportune for an emerging clinical practice. Furthermore, the moderator’s knowledge of myopia control was fundamental in order to conduct effective follow-up and constructively frame participants' responses with the discussions. We acknowledge these limitations in the Revised Manuscript as follows

*A known disadvantage of any focus group discussion is the possibility that participants may not express their honest and personal opinions about the topic. However, as focus groups can yield deeper insights into behaviour and motivations we felt this was opportune for an emerging clinical practice.  Furthermore, in a rapidly evolving field of research, focus groups provide an efficient and timely contribution of data.*

*The focus group moderator was an academic optometrist whose knowledge of myopia control was fundamental in order to conduct effective follow-up and constructively frame participants' responses with the discussions. However, pre-specified questions were used to prevent the moderator’s personal biases influencing participants' exchange of ideas and thereby generate data based on the interactions within the group discussion.*

**Reviewer comment 11**

Results:

‘Practice-based’ and ‘clinic based’ is used in the results, but community optometrists used earlier. Be consistent in naming optometric groups.

**Author response 11**

Thank you for this comment. We have updated the manuscript to clinic based Optometrists throughout.

**Reviewer comment 12**

Focus group were relatively new to professionals of 1-11 year range: again, does this limit drawing conclusions about myopia management to the profession as a whole?

**Author response 12**

As stated previously focus groups are intended to gain an in-depth understanding of an idea or issue from a selected group of individuals rather than from a statistically representative sample of a broader population. ^21^ We have highlighted this in our discussion

*Results cannot be interpreted as reflecting the awareness of all Optometrists in Ireland.*

And in the conclusion

*Although these focus groups cannot be assumed to be entirely reflective of clinical optometry practice*

**Reviewer comment 13**

Surely a barrier that should be discussed is the availability of myopia control therapies/lenses. In the introduction, the authors note that there are numerous available therapies – but then availability is highlighted as a clinical practice barrier in the Figure. However, this is not discussed in the text. Are optometrists unaware of what to order? Are they not available/distributed in Ireland?

**Author response 13**

Thank you for this comment. We have updated the Revised Manuscript to clarify what myopia control options are available in Ireland

*Low dose atropine is not readily available as a licensed product in Europe but is accessible through clinical trials, with the recruitment of trial participants dependent on optometrist referrals.* ^14–16^ *A number of contact lens options for managing myopia are also commercially available in Europe and are reported to slow myopia progression by approximately 30% to 60%.* ^8^ *. These include multifocal contact lenses, frequently fitted ‘off- label’,* ^17^ *novel myopia control lenses (becoming more readily available globally),* ^18^ *and orthokeratology contact lenses.*

The Revised Manuscript also acknowledges the perceived unavailability of myopia control therapies in the discussion

*Clinic based optometrists identified inadequate and insufficient education and training as well as a lack of availability of myopia control therapies as a fundamental justification for their reluctance to practise myopia control. This supports the results from Wolffsohn et al.’s survey that revealed, amongst other barriers, a lack of availability of and information on myopia control means that the majority of international practitioners still prescribe single-vision correction as the primary mode of management for myopic patients, even in countries where the control of myopia progression has become an important clinical goal.* ^22^ *The perceived lack of availability of myopia control management options highlights the need for further education and regulatory guidance.*

**Reviewer comment 14**

Overall I am not persuaded that the authors have answered their research question with the methodology employed. They sought to “elucidate the current practice, knowledge gaps and attitudes of optometrists in Ireland toward myopia control, and thereby identify perceived barriers that may limit the transition to a risk-focussed model of myopia management in primary care practice”. The lack of information in the way in which community optometrists in Ireland were recruited (and the small sample size) means that this work does not probably capture the current practice and knowledge gaps for the optometry profession in Ireland and likely is a biased sample. This is underlined in the relatively small number of years of experience of the focus group members, and the clear bias of interest in myopia control suggests that these optometrists are not necessarily typical of the optometric workforce.

There is interesting work here, but the authors need to make significant revision to their manuscript, fully describing their recruitment and focus group methodology and acknowledge the limitation of their study design to address their research question.

**Author response 14**

As discussed previously, we do not seek to explore a representative cross section of views from the entire profession, but rather the most informed views of interested practitioners so that any barriers that do exist can be addressed. The study was designed to explore attitudes and barriers to myopia management in practice. In order to answer this research question and to adequately explore barriers, optometrists with an interest and/or experience in myopia management are the ideal source of information. Our small sample is typical of focus groups and purposefully allowed participants to discuss their opinions and contribute in the exploration of this area. ^23^ Other barriers may exist, including a lack of interest in adopting myopia control strategies, and a wider exploration of optometrist views across the profession could offer some additional insights. These points are now highlighted in the Revised Manuscript

*Clinician personal attitudes and beliefs will also influence practices, ^47^ and although optometrists in our study appeared to have an interest in incorporating myopia control therapies into their clinical practice, study participation may be influenced by their interest in the topic, and thus results cannot be interpreted as reflecting the awareness of all optometrists in Ireland. Other barriers may exist, such as a lack of interest in adopting myopia control strategies, which could be explored across the profession, however our focus groups purposefully allowed optometrists with an interest and/or experience in myopia management to discuss their opinions and contribute in the exploration of this fast evolving area.* 

**References**

1.        Holden B, Sankaridurg P, Smith E, Aller T, Jong M, He M. Myopia, an underrated global challenge to vision: where the current data takes us on myopia control. *Eye (Lond)*. 2014. doi:10.1038/eye.2013.256

2.        Wolffsohn JS, Calossi A, Cho P, et al. Global trends in myopia management attitudes and strategies in clinical practice – 2019 Update. *Contact Lens Anterior Eye*. 2020;43(1):9-17. doi:10.1016/j.clae.2019.11.002

3.        McCrann S, Flitcroft I, Lalor K, Butler J, Bush A, Loughman J. Parental attitudes to myopia: a key agent of change for myopia control? *Ophthalmic Physiol Opt*. 2018;38(3):298-308. doi:10.1111/opo.12455

4.        Mountjoy E, Davies NM, Plotnikov D, et al. Education and myopia: assessing the direction of causality by mendelian randomisation. *BMJ*. 2018;361(k2022). doi:10.1136/bmj.k2022

5.        Huang H-M, Chang DS-T, Wu P-C, Held R, Yap E, Chia K. The Association between Near Work Activities and Myopia in Children?A Systematic Review and Meta-Analysis. *PLoS One*. 2015;10(10):e0140419. doi:10.1371/journal.pone.0140419

6.        Group. C of MET 2 SG for the PEDI. Progressive-addition lenses versus single-vision lenses for slowing progression of myopia in children with high accommodative lag and near esophoria. *Invest Ophthalmol Vis Sci*. 2011;52(5):2749-2757. doi:10.1167/iovs.10-6631

7.        Huang J, Wen D, Wang Q, et al. Efficacy Comparison of 16 Interventions for Myopia Control in Children A Network Meta-analysis. *Ophthalmology*. 2016;123(4):697-708. doi:10.1016/j.ophtha.2015.11.010

8.        Wildsoet CF, Chia A, Cho P, et al. IMI – Interventions for Controlling Myopia Onset and Progression Report. *Investig Opthalmology Vis Sci*. 2019;60(3):M106. doi:10.1167/iovs.18-25958

9.        Siu Yin Lam C, Chun Tang W, Yan-yin Tse D, et al. Clinical science Defocus Incorporated Multiple Segments (DIMS) spectacle lenses slow myopia progression: a 2-year randomised clinical trial. *Br J Ophthalmol*. 2020;104(3):363-368. doi:10.1136/bjophthalmol-2018-313739

10.      Goss DA, Grosvenor T. Rates of childhood myopia progression with bifocals as a function of nearpoint phoria: consistency of three studies. *Optom Vis Sci*. 1990;67(8):637-640.

11.      Cheng D, Schmid KL, Woo GC, Drobe B. Randomized Trial of Effect of Bifocal and Prismatic Bifocal Spectacles on Myopic Progression. *Arch Ophthalmol*. 2010;128(1):12. doi:10.1001/archophthalmol.2009.332

12.      Hasebe S, Ohtsuki H, Nonaka T, et al. Effect of Progressive Addition Lenses on Myopia Progression in Japanese Children: A Prospective, Randomized, Double-Masked, Crossover Trial. *Investig Opthalmology Vis Sci*. 2008;49(7):2781. doi:10.1167/iovs.07-0385

13.      Flanagan SC, Saunders KJ. *Optometry in Practice The Optometric Management of Childhood Myopia: A Review of the Evidence*. Vol 20.; 2019.

14.      Wu P-C, Chuang M-N, Choi J, et al. Update in myopia and treatment strategy of atropine use in myopia control. *Eye*. June 2018:1. doi:10.1038/s41433-018-0139-7

15.      McCrann S, Flitcroft I, Strang NC, et al. Myopia Outcome Study of Atropine in Children (MOSAIC): an investigator-led, double-masked, placebo-controlled, randomised clinical trial protocol. *HRB Open Res*. 2019;2:15. doi:10.12688/hrbopenres.12914.2

16.      Azuara-blanco A, Logan N, Strang N, et al. Low-dose (0.01%) atropine eye-drops to reduce progression of myopia in children: a multicentre placebo-controlled randomised trial in the UK (CHAMP-UK)—study protocol. *Br J Ophthalmol*. 2019:Epub ahead of print. doi:doi:10.1136/ bjophthalmol-2019-314819

17.      Jones L, Drobe B, González-Méijome JM, et al. IMI – Industry Guidelines and Ethical Considerations for Myopia Control Report. *Investig Opthalmology Vis Sci*. 2019;60(3):M161. doi:10.1167/iovs.18-25963

18.      The College of Optometrists. *Guidance for Optometrists- Myopia Management*.; 2019.

19.      Wolffsohn JS, Flitcroft DI, Gifford KL, et al. IMI – Myopia Control Reports Overview and Introduction. *Investig Opthalmology Vis Sci*. 2019;60(3):M1. doi:10.1167/iovs.18-25980

20.      NEJM Catalyst. What Is Risk Management in Healthcare? *Catal Carryover*. 2018;4(2). doi:10.1056/CAT.18.0197

21.      O.Nyumba T, Wilson K, Derrick CJ, Mukherjee N. The use of focus group discussion methodology: Insights from two decades of application in conservation. Geneletti D, ed. *Methods Ecol Evol*. 2018;9(1):20-32. doi:10.1111/2041-210X.12860

22.      Wolffsohn JS, Calossi A, Cho P, et al. Global trends in myopia management attitudes and strategies in clinical practice. *Contact Lens Anterior Eye*. 2016;39(2):106-116. doi:10.1016/j.clae.2016.02.005

23.      Krueger RA, Casey MA, Donner J, Kirsch S, Maack JN. Social Analysis: Selected Tools and Techniques. *World Dev*. 2001;(36). doi:10.2165/00019053-200725120-00005
